# A Normalization Model of Attentional Modulation of Single Unit Responses

**DOI:** 10.1371/journal.pone.0004651

**Published:** 2009-02-27

**Authors:** Joonyeol Lee, John H. R. Maunsell

**Affiliations:** 1 Department of Neuroscience, Baylor College of Medicine, Houston, Texas, United States of America; 2 Howard Hughes Medical Institute and Department of Neurobiology, Harvard Medical School, Boston, Massachusetts, United States of America; Victoria University of Wellington, New Zealand

## Abstract

Although many studies have shown that attention to a stimulus can enhance the responses of individual cortical sensory neurons, little is known about how attention accomplishes this change in response. Here, we propose that attention-based changes in neuronal responses depend on the same response normalization mechanism that adjusts sensory responses whenever multiple stimuli are present. We have implemented a model of attention that assumes that attention works only through this normalization mechanism, and show that it can replicate key effects of attention. The model successfully explains how attention changes the gain of responses to individual stimuli and also why modulation by attention is more robust and not a simple gain change when multiple stimuli are present inside a neuron's receptive field. Additionally, the model accounts well for physiological data that measure separately attentional modulation and sensory normalization of the responses of individual neurons in area MT in visual cortex. The proposal that attention works through a normalization mechanism sheds new light a broad range of observations on how attention alters the representation of sensory information in cerebral cortex.

## Introduction

Attention to a visual stimulus can greatly influence the responses of individual neurons in visual cortex (see [1]–[3]). Descriptive models like the biased competition model and the feature similarity model successfully describe various aspects of the effects of attention on sensory responses, but provide little insight into the mechanisms by which attention's effects are mediated. Here we propose a model that extends previous descriptions of attentional modulation by linking attentional modulation to neuronal mechanisms that have been described in mediating sensory response normalization.

Sensory normalization is a form of gain control in which neurons' responses are reduced in proportion to the activity of large pools of neighboring neurons. Because normalization has a divisive effect on all of a neuron's responses, it scales responses without altering stimulus preference or stimulus selectivity, providing a pure form of gain control. Normalization models were introduced to explain nonlinearities in the responses of V1 simple cells, such as the sigmoidal shape of their contrast response functions (CRFs) and the inhibitory effect of adding a second stimulus to the receptive field at a non-preferred orientation [4]–[7]. Subsequent studies showed that a similar kind of normalization could explain the nonlinear response properties of other visual areas, including the middle temporal area (MT) [6], [8], [9] and inferotemporal cortex (IT) [10]. Normalization has also been put forth as a mechanism to reduce redundancy in the neuronal representation of natural stimuli [11].

Certain findings from previous studies of attention suggest that the neuronal mechanisms that underlie its effects on visual neurons might be closely related to the type of gain control mediated by response normalization. When attention is shifted toward or away from a stimulus in the receptive field of a neuron, it causes a multiplicative scaling of tuning curves for stimulus orientation, direction, or contrast [12]–[15]. A normalization mechanism would be well suited to mediate a multiplicative scaling. Additionally, the modulation of a neuron's responses is typically much stronger when attention is shifted between two stimuli within its receptive field, compared to shifting attention toward or away from a single stimulus in the receptive field [16]–[18]. This difference would be expected if normalization mechanisms were involved, because the effects of normalization can be greatly reduced when only one stimulus is present.

We propose that attention is not only related to neuronal response normalization mechanisms, but may depend on them. We suggest that the primary effect of attention in visual cortex is to modulate the strength of normalization mechanisms. We refer to this concept as “attentional normalization”. We present here a model of attentional normalization and show that it can readily account for a wide variety of attentional effects described in visual cortex. We demonstrate that this simple model can explain the way neurons in monkey visual cortex respond to both stimulus interactions and changes in attention. These observations have the potential to help clarify a broad range of observations about attentional effects on sensory responses in cerebral cortex. Some of these findings have been previously presented in abstract form [19].

## Results

### Attentional normalization model

Response normalization models (e.g., [4]) typically assume that the response of a given cell depends on a linear receptive field, divisive normalization, and non-linear spiking threshold. When a stimulus falls on the receptive field of a neuron, the linear receptive field produces a tuned output, which determines the neuron's selectivity for properties such as orientation, direction, spatial frequency, or temporal frequency. Additionally, each stimulus activates a pool of neurons whose receptive fields overlap with the stimulus, and the summed activity of this pool acts to reduce the activity of the neuron under consideration (and other neurons driven by the stimulus) by dividing its response in proportion to the pool's summed activity. The normalized signal then passes through the nonlinear threshold stage, producing an output rate of firing. An important assumption is that the strength of the divisive normalization signal is unaffected by stimulus properties such as orientation or spatial frequency because it comes from a population of cells of varying tuning properties [20], and a stimulus of any value would activate a comparable number of neurons. Thus, the strength of the normalization is typically assumed to depend only on the contrast of the stimulus.

One of nonlinearities of neuronal responses in primary visual cortex (area V1) is a phenomenon called “cross orientation inhibition.” If a stimulus with a preferred orientation falls on the receptive field of a V1 neuron, and a second stimulus with a non-preferred orientation is superimposed, the response of the cell is inhibited by the non-preferred stimulus. This happens even if the non-preferred stimulus by itself causes no response or is somewhat excitatory. Response normalization models explains this phenomenon because the second stimulus increases the amount of divisive normalization. Carandini et al. [7] demonstrated the success of the normalization model for explaining cross orientation inhibition. They used a model with the following form to explain the responses of V1 neurons to plaid stimuli made of two superimposed sinusoidal gratings:
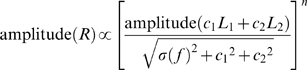
(1)


In the equation, 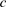 is the contrast of the gratings, 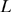 is the response of the linear receptive field to each grating at unit contrast, 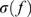 is the semi-saturation parameter as a function of temporal frequency of the grating, and 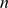 is the exponent for the nonlinear threshold stage. They assumed that the untuned normalization was proportional to contrast of stimuli, so this equation effectively computes a weighted average of the responses to the two gratings, with each grating weighted by its contrast. This implementation explains not only neuronal responses to a single stimulus but also the effect of stimulus interactions when multiple stimuli are present.

We adopted the general approach of Eq. 1 to implement a model of how attention modifies neuronal responses. We simplified the implementation somewhat, because our primary goal was to model the effect of attention, but not to model details of the neuronal responses to stimuli. We therefore took responses to individual stimuli as a given from empirical observations, and focused on stimulus interactions and effects of attention. We followed the approach of Britten and Heuer [21] in modeling how neurons response to pairs of stimuli. They modeled response summation of neurons in area MT of rhesus monkeys using a power-law equation:
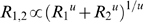
(2)



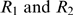
 are the responses to two stimuli when they appear individually in the receptive field of an MT neuron, and 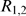 is the response expected when they appear together. A subsequent study showed that a power-law of this kind is useful for explaining the nonlinear response summation of neurons in area V4 [22].

**Figure 1:**
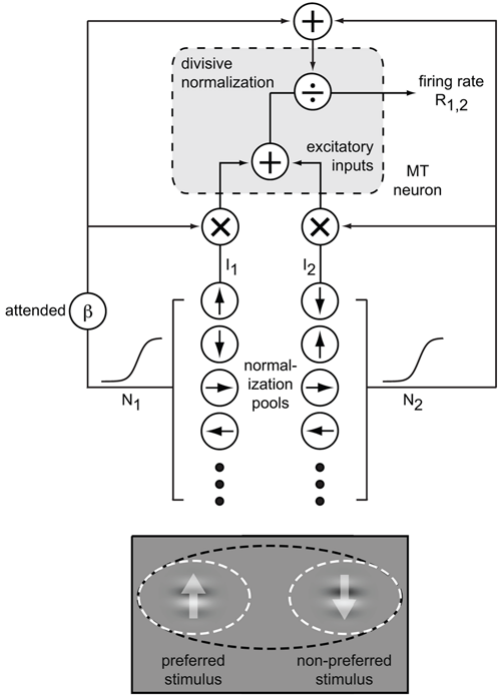
Attentional normalization model. The black dotted ellipse shows the receptive field of the neuron being considered, and the white dotted ellipses show receptive fields of neurons that provide input to this neuron. Each set of input neurons constitutes one normalization pool. Arrows overlapped with Gabors show the motion direction of the drifting Gabors. See text for details.

We present here a model that explains responses when two stimuli are present in a neuron's receptive field, however, this model can be easily extended to treat any number of stimuli. We assume that a neuron with a receptive field containing two stimuli receives a direct, tuned input with a strength that depends on how well the stimulus matches the preferred stimulus for the cell (Figure 1). The cell also receives divisive normalization inputs from two populations of neurons, each activated by one of the stimuli. The normalization terms associated with each stimulus contribute to producing an overall response that is a weighted average of the direct inputs, which is similar to Eq. 1. Equation 3 describes the response of a neuron with two stimuli in its receptive field.
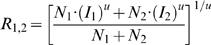
(3)


In this equation, 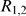 is the response of the neuron, 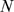 is the normalization term for each stimulus, 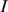 is the direct input driven by each stimulus, and 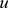 is a power term that accommodates the nonlinear summation of the two inputs (as in Eq. 2). Because the magnitude of each normalization signal depends on the contrast of the associated stimulus, we let the strength of the normalization be an exponential function of the contrast of that stimulus.
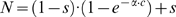
(4)


This normalization signal takes values from 0 to 1 and has two free parameters of 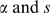, where 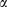 is the slope of normalization, 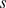 is the baseline level of normalization. The contrast of the stimulus is given by 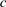. The term 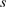 plays a role that is similar to the 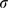 term of Eq. 1. It remains when the contrast of the stimulus is zero and prevents the response from becoming infinite. Equation 4 describes the normalization contributed by each of the stimuli in the receptive field. Heuer and Britten [23] developed a similar model for explaining contrast dependent response summation of neurons in area MT. Their normalization term was a hyperbolic ratio function of contrast, which has been shown to do a good job of describing how contrast affects the responses of cortical neurons [23], [24]. We instead used an exponential function for normalization because it produced qualitatively indistinguishable results with one fewer free parameter (explaining a 94.9% versus 95.3% of the variance in the mean responses of neurons; see below).

Although the normalization functions for the two stimuli have the same parameters, they take different values when the stimuli have different contrasts. The direct inputs are each multiplied by their respective normalization terms, following the form of Eq. 1. This multiplication, coupled with a division by the summed normalization inputs, has the effect of making the response of the neuron a weighted average of the direct inputs.

The effect of attention is introduced by letting attention modulate the normalization associated with the attended stimulus. We extend Eq. 4 by adding an attention term, 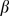, which is 1 for unattended stimuli but can take other (typically larger) values for attended stimuli:
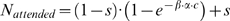
(5)


In this way, attention acts only through the normalization mechanism. We were motivated to take this approach because attention frequently produces a multiplicative gain of neuronal responses. With this approach, the effects of changing stimulus contrast or of changing attention will be similar in that both affect normalization mechanisms to modulate the neuronal response, but they will differ in that changes in attention, unlike changes in stimulus contrast, will not change the direct inputs (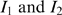 in Figure 1). Thus, the attentional normalization model dissociates the effect of changing stimuli parameters from the effects of changing the locus of attention.

In the following sections we show that the attentional normalization model accounts for key observations about the way that both attention and nonlinear stimulus summation changes neuronal responses, using previously published data and data from experiments we have performed.

### Simulation for a response summation

**Figure 2:**
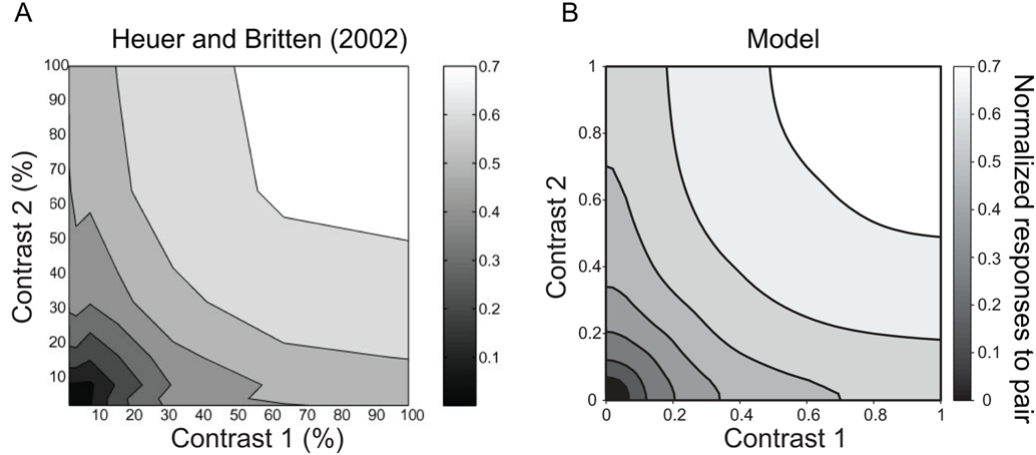
Data and simulations for contrast dependent response summation. A: Average responses of MT neurons to pairs of stimuli that had independent contrasts. Reproduced with permission from Figure 9 of Hilary W. Heuer and Kenneth H. Britten, Contrast dependence of response normalization in area MT of the rhesus monkey. J. Neurophysiol. 88:3398–3408, 2002. Each cell was normalized by its maximum firing rate, and responses were averaged across 39 MT neurons. B: A contour surface showing a simulation of this test using the normalization model. The plot is generated by averaging predicted responses of 100 model neurons.

We first tested whether the attentional normalization model can replicate physiological data by simulating response summation and comparing the result with physiological data obtained in area MT. Figure 2A plots population data from a study by Heuer and Britten [23], in which they measured response summation by placing two stimuli with preferred directions of motion at non-overlapping locations in the receptive field of each neuron. The axes show the contrast of each stimulus and contours follow iso-response lines. On this plot, when the contrasts are relatively high (>40%), the response contours are concave. However, when the contrasts are low (<30%) they are convex.

We simulate responses to pairs of stimuli by vary the contrasts of two stimuli independently in the attentional normalization model. For the simulation, we assumed that two equally-effective stimuli (e.g., preferred) fell on different locations in the receptive fields of MT neurons. We simulated responses to individual stimuli and paired stimuli at different locations in the receptive field, and used parameters from physiological data for the simulation (see Methods). Responses of each neuron were normalized by its maximum firing rate, and they were averaged across neurons (see Methods). Figure 2B is the contour surface resulting from the simulation. It shows the same concavity and convexity as the MT recordings. Thus, the attentional normalization model replicates the effect of contrast dependent normalization on response summations of MT neurons.

### Simulation of attention with a single stimulus in the receptive field

One property of attention is that it can change the magnitude of a neuron's response without appreciably affecting its selectivity. When attention is directed toward or away from a single stimulus in the receptive field, tuning curves for orientation, direction, and contrast are scaled vertically, without appreciable changes in the preferred stimulus or the breadth of tuning [13]–[15]. Thus, the primary effect appears to be a change in the gain of a neuron's response to all stimuli. Because attention acts on a divisive (multiplicative) term in the attentional normalization model, this model appears well suited to explain this behavior.

We simulated a single stimulus by setting the contrast of the second stimulus to zero in Eq. 3. This does not completely remove the normalization term for this stimulus, because the normalization has a non-zero baseline level of activity (Eq. 4). Consequently, the normalization terms do not drop out of Eq 3, and attention can affect responses. The remaining normalization allows attention to modulate responses to the single stimulus because it affects the relative weight of the remaining direct input. The form of the model presented here considers only two stimuli, so when no stimuli are present (0% contrast), two units of spontaneous activity contribute to the normalization. This might appear arbitrary, because there might be an infinite number of stimuli that do not appear. Increasing the number of stimuli considered will change the relative weight of the direct inputs and the normalization inputs, such that the response to any number of zero-contrast stimuli will be the same as the response to two zero-contrast stimuli. Changing the number of receptive field stimuli considered redefines the parameters of the model without changing its other properties (see Methods).

**Figure 3:**
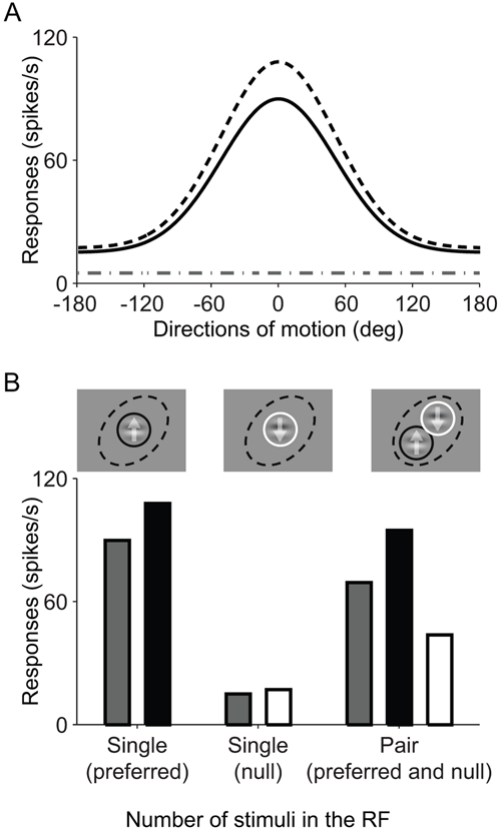
Simulations of effects of attention. A: Simulation of an attention effect on a direction tuning function. The solid black line is the direction tuning function with attention directed away from the stimulus, and the dotted black line is the tuning function with attention directed toward the stimulus. The dashed-dotted line is the spontaneous activity of the model neuron. B: Simulation of the effects of attention on responses to single and paired stimuli. The Gabor with the up-arrow is the preferred stimulus and the Gabor with the down-arrow is the non-preferred stimulus. The black dotted ellipse shows the receptive field, and the black and white circles mean attention to the preferred stimulus and non-preferred stimuli, respectively. Grey histograms are the responses when attention is directed to a stimulus outside the receptive field (not shown). Black and white histograms are the responses when the attention is directed to the preferred stimulus or the null stimulus in the receptive field, respectively. For simulations here, we used parameters of 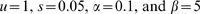.

Because responses to a single stimulus are needed as input for the attentional normalization model, we simulated responses of MT neurons to different directions of 100% contrast motion stimuli (e.g., drifting Gabor or random dot patch) with a Gaussian function (see Methods). We then simulated the effect of attention on direction tuning functions. As expected, the attentional normalization model readily accounts for the multiplicative scaling effect of attention. Figure 3A shows the result of a simulation using the attentional normalization model. The solid line is the tuning function without attention, and the dashed line is the tuning function when the attention is directed to the stimulus in the receptive field.

The effect of attention shown in Figure 3A is a change in response gain. Regardless of the stimulus direction, the response (activity above spontaneous activity) is increased by the same factor. However, when no stimulus is present, attention does not affect spontaneous activity. A proportional scaling of all activity, including spontaneous activity, is termed activity gain. Response gain has been described in some experiments [25], but activity gain is more commonly reported, especially for spatial attention [15], [26], [27]. Failure to affect spontaneous activity is a known limitation of the current model. Additional terms or reconfiguration of the model could obviously fix this, but we have not pursued this minor deficiency because spontaneous activity is typically a very small component of neuronal activity so it has little effect on the model's overall performance. In the future we plan to collect neurophysiological data that are sufficiently precise that they could guide a specific modification of this aspect of the model.

### Simulation with a pair of stimuli in the receptive field

Although the primary effect of attention has been described as a gain change, stronger and more complicated effects are seen when attention is shifted between two stimuli in a neuron's receptive field. Very strong modulations are typically seen when attention is shifted between a preferred and a non-preferred stimulus that both lie within the receptive field [16], [17], [22], [26]. These effects have been described as a shrinking or shifting of the receptive field that weights its responses toward inputs corresponding to the attended stimulus. This effect cannot be explained as a simple change in neuronal sensitivity because it involves a change in the spatial weighting of a receptive field: the neuron becomes more responsive to one portion of visual space while becoming less responsive to another.

The normalization that exists when more than one stimulus is in the receptive field can explain this effect of attention. If attention is directed to one of two (or more) stimuli in the receptive field, it will adjust the weights of inputs from each stimulus by changing the strength of their respective normalization signals. Therefore, the response of the cell will increase or decrease depending on the efficacy of the attended stimulus. In addition, the size of attentional modulation with one stimulus in the receptive field will be smaller than the modulation with two stimuli in the receptive field because the strength of normalization that cell receives is proportional to the number of stimuli in the receptive field (and the weights of inputs are determined by the normalization, Eq. 3).

To test whether the attentional normalization model explains effects with two stimuli inside the receptive field, we simulated this configuration and observed how the neuronal response changed as the locus of attention changed. We simulated a pair of preferred and the non-preferred stimuli at 100% contrast. Presented individually, the responses to these stimuli corresponded to the peak and trough of the tuning curve in Figure 3A. Figure 3B shows the behavior of the model neuron. The first two bars show the model predictions for a single preferred stimulus, with and without attention. The second two bars show the predictions for a single non-preferred stimulus, with and without attention. The final set of bars shows responses with the preferred and non-preferred stimuli both inside the receptive field. The gray bar show the model's response when the attention is directed outside the receptive field, the black bar show the response when attention is directed toward the preferred stimulus in the receptive field, and the white bar show the response when attention is directed toward the non-preferred stimulus in the receptive field. These predictions of the attentional normalization model are consistent with neurophysiological observations. Directing attention to the preferred stimulus increases the response relative to when attention is directed outside of the receptive field, and directing attention to the non-preferred stimulus decreases the response. The amount of attentional modulation resulting from shifting attention between preferred and non-preferred stimuli in the receptive field is greater than directing attention toward or away from a single stimulus in the receptive field. The size of attentional modulation when attention is switched between preferred and non-preferred stimuli in the receptive field, was about 6 times greater than it when attention is switched between single preferred stimuli inside and outside of the receptive field under the given parameters of the model (see Figure legends for parameters).

### Attention and stimulus interaction

**Figure 4:**
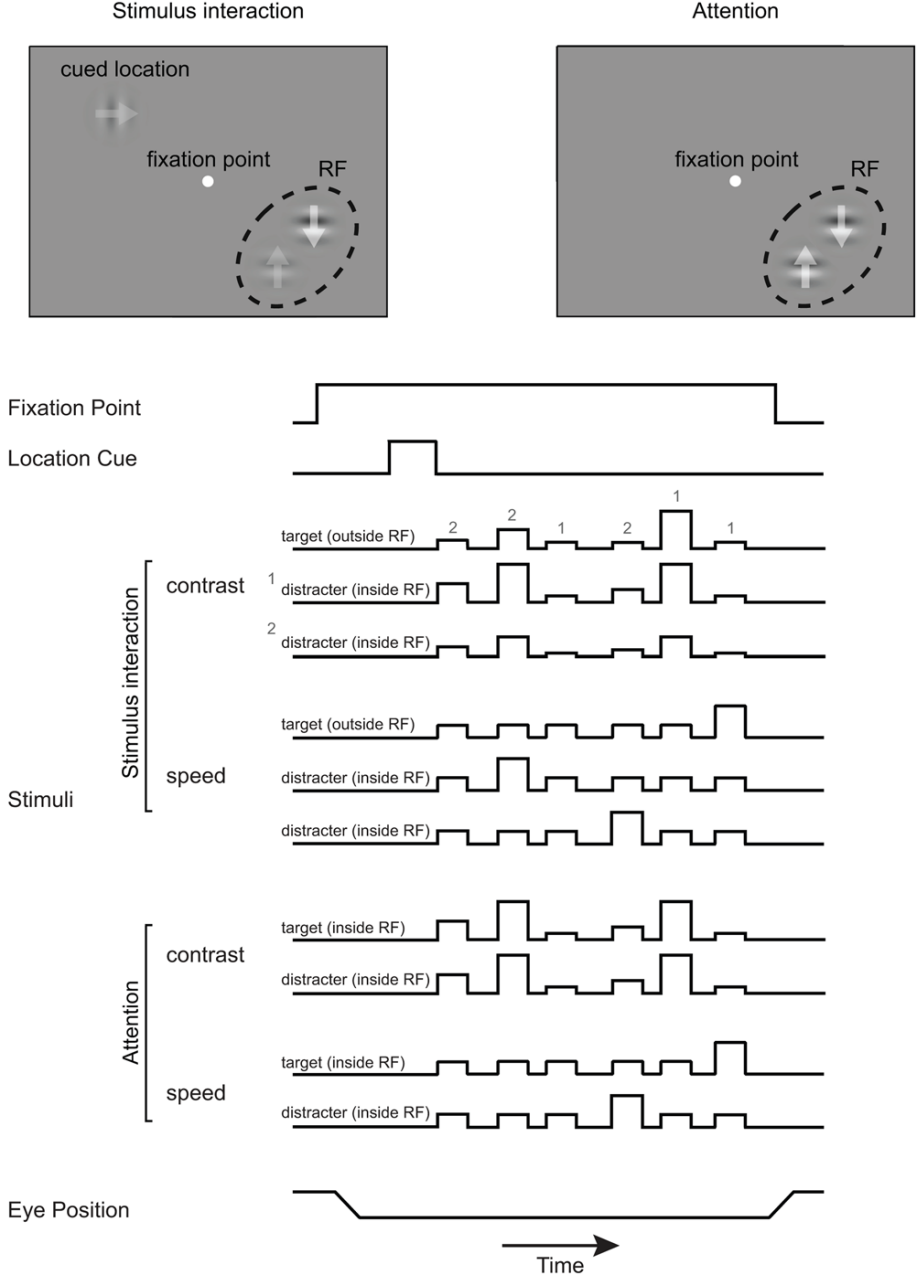
Task designs for the stimulus interactions and attention experiments. The top panels show the stimulus configurations for the two experiments and the diagrams below show the structure of individual trials. The arrows overlapped the Gabors show the directions of their motion. The height of the lines in the trial plots represents the contrast or speed of the Gabor in each stimulus location. In the stimulus interactions experiment, the contrasts of the Gabor in one location are either twice or half the contrasts of the Gabor in the other location in the receptive field. Grey digits show that the contrasts of the target stimulus are randomly selected from the contrasts of two distracters in the receptive field. In the attention experiment, the contrasts of the two stimuli in the receptive field are matched with each other (see Methods).

Additional support for the normalization model is provided by quantitative assessment of its ability to simultaneously account for neurophysiological responses to both changes in stimulus contrast and changes in the focus of attention. This analysis was based on responses that were recorded from MT neurons in a rhesus monkey for a different purpose. The monkey did two tasks that made it possible to measure attentional modulation and stimulus interactions independently (Figure 4). To measure attentional modulation, pairs of drifting Gabors were presented in the receptive field. The Gabor in one location always drifted in the neuron's preferred direction and the Gabor in the other location always drifted in the opposite (null) direction. The contrasts of the two Gabors were always matched, but they varied from presentation to presentation, and on different trials the animal's attention was directed to one or the other Gabor (see Methods). In this way we could measure how the CRF of the cell varied as attention was shifted from the preferred stimulus to the null stimulus. To measure stimulus interactions, attention was kept constant by adding drifting Gabors at a third location far outside the receptive field and directing the animal's attention to that location on every trial. While the animal's attention was directed away from the receptive field, the preferred and null stimuli within it were presented with a range of contrasts, but with the contrast of the preferred and null stimuli always differing by a factor of two.

**Figure 5:**
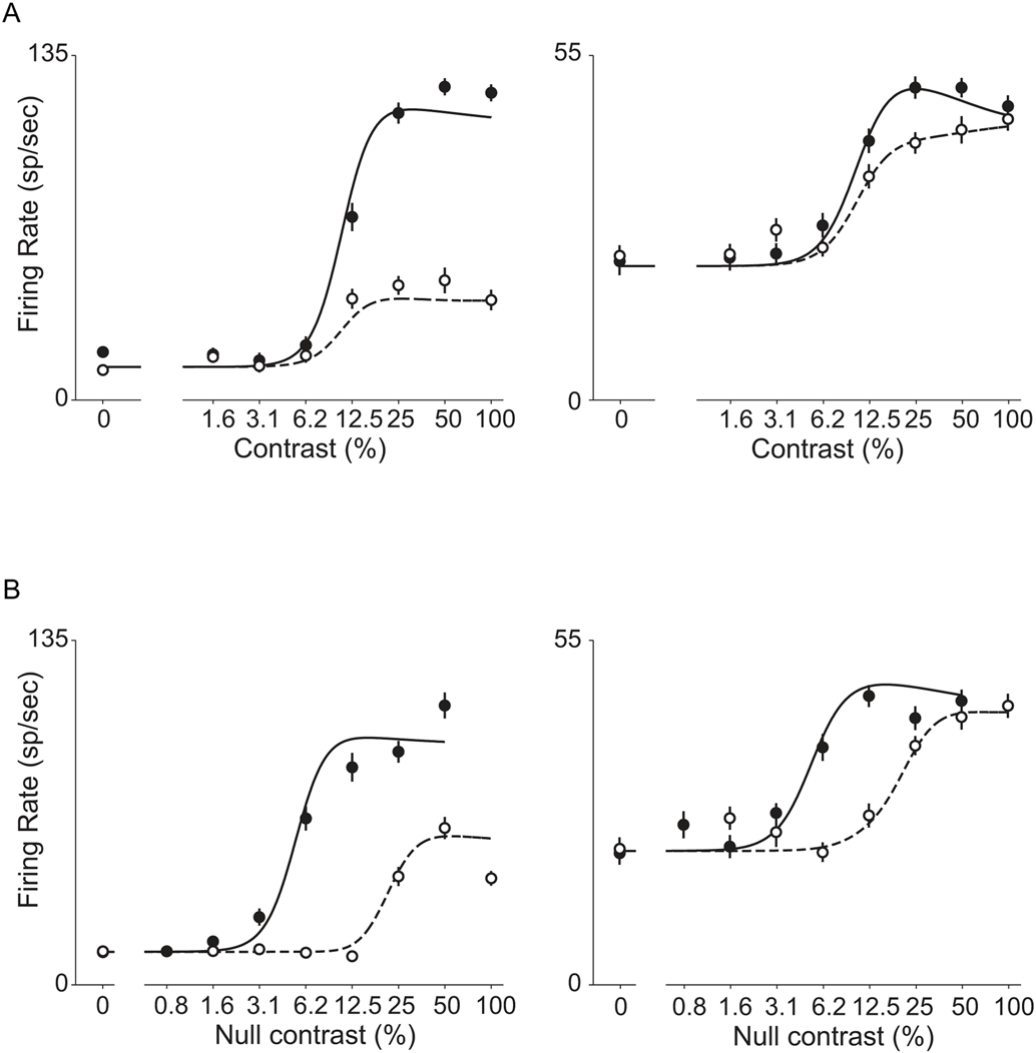
Contrast response functions for two example neurons from attention and stimulus interactions experiments. A: Responses of each neuron in the attention experiment. Filled circles are responses when attention is directed to the preferred stimulus and open circles are responses when attention is directed to the null stimulus. Solid and dotted lines are the best fitting functions of the attentional normalization model. B: Responses of each neuron in the stimulus interactions experiment. Filled circles are responses when the preferred stimulus has a higher contrast than the null stimulus (2×), and open circles are responses when the null stimulus has a higher contrast than the preferred stimulus (2×). Solid and dotted lines are the best fitting functions of the model. Vertical lines are standard errors and the best fitting functions were obtained by fitting data from the attention and stimulus interactions experiments simultaneously to the attentional normalization model.

The panels in Figure 5A show the responses of two example MT neurons to the attention experiment. In both attention conditions, the example neurons had characteristic sigmoidal CRFs, but the responses were stronger while the animal was paying attention to the preferred direction (filled symbols, solid lines) relative to when the same stimuli appeared but the animal's attention was directed toward the null stimulus (open symbols, dashed lines). The responses of the cell in the left panel were strongly modulated by the stimulus to which attention was directed, while the responses of the cell at the right panel were weakly modulated by attention. The effect of attention on the contrast tuning functions is reasonably well described as a gain change, similar to the effect seen with orientation or direction tuning [13], [14]. The gain change was response gain, in which only the driven portion of the activity was modulated by attention, rather than an activity gain, in which the spontaneous activity (0% contrast) is also modulated [see 15]. The spontaneous activity did not change between the two conditions because in both cases the animal was attended to (different) locations inside the receptive field. Changes in spontaneous activity have only been reported when attention is shifted from a location inside a neuron's receptive field to a location outside the receptive field studies[15], [26]. The panels in Figure 5B shows how the same cells responded when attention was kept constant (toward the stimulus far outside the receptive field) and the preferred stimulus was presented at either twice or half the contrast of the null stimulus. The responses in Figure 5B are plotted as a function of the null stimulus contrast. The filled symbols show responses recorded when the preferred stimulus had twice the contrast of the null stimulus (e.g., preferred 50%, null 25%). The open symbols show the responses recorded when the preferred stimulus had half the contrast of the null stimulus (e.g., preferred 25%, null 50%). The horizontal offset between the two sigmoid functions is expected because the preferred stimulus dominates the response, and at any contrast of the null stimulus, the preferred stimulus had four times more contrast in one condition compare to the other. Consistent with this, the horizontal offset between the rising phases of the two curves is close to a factor of four on the contrast axis (which has log scaling). In addition to this horizontal offset, there is a vertical offset between the upper saturation of the two functions for the responses in the left panel. This vertical offset arises from response normalization, as will be explained later.

The capability of the attentional normalization model to account for both the attentional modulations and the stimulus interactions was demonstrated by fitting the responses of each neuron to the attentional measurements and the stimulus interaction measurements simultaneously to the model. The model has four free parameters (Eqs. 3, 4, and 5: 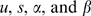) and the number of data points for each cell used here was 30 (8 contrast×2 attention conditions = 16 from attention experiment, and 8 contrast×2 stimulus conditions−2 spontaneous activity = 14 from stimulus interaction experiment). The solid and dashed lines in Figure 5A and B show the fits the model provides for the two example neurons. Using a single set of parameters for each cell, the attentional normalization model does an excellent job of accounting for the effects of both varying attention when relative contrast is fixed and of varying relative contrast when attention is fixed. The model explains 97% and 96% of the variance of the mean responses of these two cells. Across all the cells tested (n = 25), the median of the variance explained by the fit was 95%.

One of the example cells (Figure 5, left panels) was strongly modulated by attention and also showed a pronounced vertical offset in the upper saturations of the CRFs during the stimulus interaction measurements. The other cell (Figure 5, right panels) was weakly modulated by attention and showed little vertical offset in the upper saturation of the CRFs during the stimulus interaction measurements. A vertical offset is expected during stimulus interaction measurements because normalization causes the higher contrast stimulus to be given more weight (Eq. 3). Responses will be stronger when the preferred stimulus is given more weight owing to greater contrast (e.g., 100% preferred, 50% null) and weaker when the null stimulus is given more weight (e.g., 50% preferred, 100% null). An offset of this sort will not happen, however, if the normalization is saturated and does not vary over the range of higher contrasts. We believe that this is the explanation for the failure to see a vertical offset for the neuron in the right panels of Figure 5.

**Figure 6:**
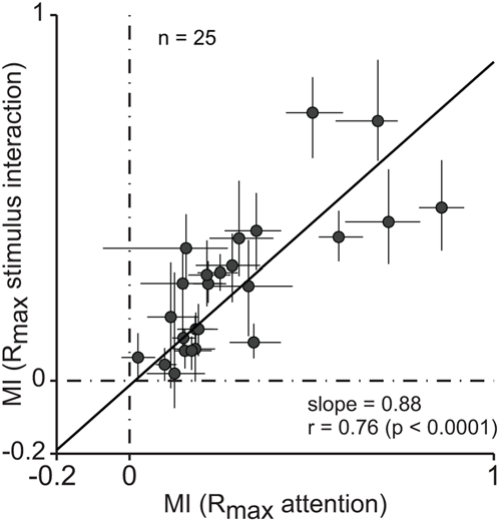
Correlations between response modulations from attention and sensory normalization. Each value in the scatter plot shows the modulation indices of individual neurons for attention and sensory normalization. Vertical and horizontal lines are 95% confidence intervals from bootstrap analyses. The regression line is the best fitting function of a linear equation (see Methods). The rho is a Pearson's correlation coefficient.

An important feature of the attentional normalization model is that because attentional modulation is constrained to act through the normalization mechanism (Figure 1), there can be no attentional modulation if the normalization is saturated and does not vary. For this reason, we expect to see little attentional modulation in those neurons that evince little response normalization. We tested whether a correlation between the strength of attentional modulation and sensory normalization exists for MT neurons. For each neuron we measured the strength of attentional modulation by taking the 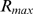 parameters from the best fitting functions (the maximum attainable responses, see Methods) and used them to compute a modulation index. We similarly took the 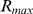 parameters for the model's fit to the stimulus interaction measurements and computed an analogous index. Figure 6 is a scatter plot of the indices, with each point corresponding to one neuron. Vertical and horizontal lines on each point are 95% confidence intervals from a bootstrap analysis (see Methods). The regression line was obtained by fitting the values to a linear equation, using a weighted least squares method (the confidence intervals from the bootstrap analysis served as weight). There was a strong correlation between the two values. Notably, the best fitting line goes through the origin (intercept of the fit: −0.01), as predicted by the attentional normalization model. If normalization cannot modulate responses, attention should be unable to produce any effect (see Figure 1). This observation would not be expected if attention could operate independently of the normalization mechanism, for example by directly changing the overall gain of all responses or by directly modulating the excitatory drive associated with inputs driven by one stimulus or the other.

It is important to note that this correlation might rise spuriously from the directionality differences (i.e., differences between responses to the preferred stimulus and responses to the null stimulus). For example, if a cell were not tuned for direction of motion such that the responses to the preferred direction and the null direction are the same, then we would expect no modulation either from shifting attention or from changing stimulus contrast, because the two stimuli would be equivalent. Thus, the correlation in Figure 6 could arise from a sample of neurons with different degrees of direction selectivity. However the correlation reported here cannot be explained this way. First, almost every MT cell shows strong direction tuning (average 11∶1, [28]), which was true for the current data (mean directionality of 15∶1). Many of the cells with strong direction tuning had little or no modulation in the stimulus interactions and attention experiments. Second, analysis of the correlations among stimulus interaction, attention, and directionality showed that directionality was not an important factor (see Methods). The relationship between sensory interaction and attention yielded the strongest pairwise correlation (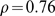, p<0.0001), and it alone remained significant when we compensated for the effect of the third variable by using partial correlations (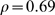, p<0.001).

## Discussion

We propose that attentional modulations in visual cortex depend on response normalization mechanisms, and have presented a basic attentional normalization model to demonstrate the feasibility of this idea. The model accounts for key observations on the effects of attention in visual cerebral cortex. Additional support for the normalization model comes from neurophysiological data showing a close relationship between the strength of stimulus interactions that are likely to be mediated by normalization and the strength of attentional modulations.

A role for normalization mechanisms in attentional modulation has been suggested by earlier studies of attention. In a psychophysical study, Lee and colleagues [29] proposed that response normalization was involved in attention-mediated changes in discrimination thresholds for luminance contrast, orientation, and spatial frequency. They found that they only could explain their results by assuming that attention changed nonlinear interactions between populations of overlapping filters, where nonlinear interactions carry out the normalization. A recent neurophysiological study found a correlation similar to the one described here, in which attentional effects were correlated with the strength of border-ownership preferences in area V2 [30]. This study is consistent with our observation in that attention uses a sensory mechanism that determines response properties of V2 neurons through indirect pathways, for example, lateral or feedback connections. Reynolds and Heeger have recently proposed a model to explain attentional modulation of responses of neurons in visual cortex that depends on normalization mechanisms [31].

### Relationship to other models of attention

While the attentional normalization model suggests a specific mechanism for how attention modulates the neuronal responses, it is not inconsistent with previous models. The most widely recognized model addressing attention is the biased competition model [1], [16]. This model suggested that when multiple stimuli are present they compete for dominance of a neuron's response, and attention biases that competition in favor of the attended stimulus. The biased competition model, as originally presented, is descriptive and does not propose a specific mechanism. While many studies have described their results as consistent with biased competition (e.g., [32]–[38]), such statements amount to a confirmation that when multiple stimuli are presented, attention to one of those stimuli brings a neuron's response closer to what it would be if that stimulus were presented alone. Because the attentional normalization model presented here is also consistent with previous observations on attentional modulations, it is consistent with biased competition, and but goes beyond to provide a mechanism that explains how attention might alter the relative influence of two or more stimuli.

Subsequent reports [3], [17] describing experiments on attention presented a specific model in the context of biased competition (although it was not presented as definitive model for biased competition). That model has some properties in common with our attentional normalization model. In particular, it implements a form of divisive normalization because inputs are divided by the sum of all inputs. However, it did not discuss sensory normalization or include normalization as a central component. Moreover, this biased competition model makes several predictions that differ from those of the attentional normalization model, and which are inconsistent with physiological data. First, it equates the effect of attention to the effect of increasing stimulus contrast, although attention and changes in contrast have distinct effects on the contrast response functions of neurons (Lee and Maunsell, submitted). Second, this biased competition model predicts that attention will not affect the upper saturation of contrast response functions for a single stimulus in the receptive field, an effect that has been shown to occur in many studies [15]. Third, this biased competition model cannot explain a correlation between sensory normalization and attentional modulation (Figure 6).

The feature similarity model of attention is another important model of attention [25], [39], [40]. It suggests that attention adjusts the gain of each neuron in proportion to the similarity between the attended feature and the neuron's preference for that feature. Spatial location is viewed as a feature, so spatial attention is simply a subset of feature attention. This model is consistent with physiological data, but it does not address mechanisms. The attentional normalization model is consistent with the feature similarity model because its mechanism predicts the effects described by feature similarity. Although we have described attentional normalization in terms of spatially-separated stimuli, it could apply equally well to attention to stimulus features, such as color or orientation. The critical requirement is that attention should be able to modulate the activity of a normalization pool that captures the attended feature, whether spatial or otherwise. This may be more of a challenge for non-spatial features. For example, if attention is directed to one of two co-extensive patches of random dots that move in different directions, the attentional normalization model can produce the expected changes in neuronal responses as long as there is a separate normalization pool for each of the different directions of motion. Whether that is the case is an empirical question that will provide an important test of the attentional normalization model.

### Underlying biophysical mechanisms

In the original model of response normalization, a shunting type of inhibition was suggested for the biophysical mechanism of normalization because it acts as a divisive factor. Previous studies have reported how shunting inhibition explains gain modulation. Some emphasize the importance of balanced excitatory and inhibitory inputs [41], [42], while others emphasize the importance of synaptic noise [43], [44] or short-term synaptic depression [12]. The current study makes no claims about underlying biophysical mechanisms for attentional modulation except that it requires some type of mechanism (biophysical or circuit) that implements divisive normalization.

Given that normalization is mediated by a pooled inhibitory mechanism, it may depend on inhibitory interneurons [45], which mainly acts as “modulator” [46]. The attentional normalization model makes no assumptions about specific cell types, however. Because the attentional normalization model affects neuronal responses through normalization, attention is expected to modulate the activity of neurons in the normalization mechanism as well as the neurons that are affected by normalization. Consistent with this idea, a study that examined the effects of attention on narrow-spiking neurons (putative interneurons) and broad-spiking neurons (putative pyramidal cells) found that attention modulated both cell types proportionately [47].

### Implications for understanding attentional modulation

The attentional normalization model could represent a valuable advance in understanding several attributes of attentional modulation. First, it explains why the primary effect of attention appears to be a gain change that does not affect the breadth of tuning curves. Because attention acts primarily through divisive normalization, its effects primarily take the form of a multiplicative scaling of tuning curves. Second, it explains why modulation can be so much stronger when attention is shifted between two stimuli in the receptive field, compared to shifting attention between a stimulus in the receptive field and a distant stimulus. Although the normalization model can produce some modulation with a single stimulus in the receptive field, the effects of attention are much more potent when it acts differentially on the normalization of different stimuli in the same receptive field.

Another aspect of attentional modulation that attentional normalization may help to explain is the variability between neurons in the amount of attentional modulation they express, both within and between areas. It is a common observation that different neurons show more or less attentional modulation, even when task demands are kept as constant as possible [48]. The source of this variance has not been explained. If attentional modulation depends on response normalization mechanisms, it is possible that the variance in attentional modulation is a consequence of variance in normalization, arising either from inherent variations in the strength of normalization from cell to cell, or subtle differences in stimulus configurations that cause normalization to vary for tests of different neurons [49]. Similarly, it is a common observation that attentional modulation grows stronger in later stages of visual cortex. The source of this variance is similarly unknown. The attentional normalization model suggests that it may depend more on changes related to sensory normalization, such as differences in receptive field size or the need to remove redundancy in sensory coding [11], than on differences in the strengths of inputs from higher centers.

### Limitations of the current model

Although the attentional normalization model explains many aspects of attentional modulation, it remains incomplete. In the current version, attention modulates normalization by changing the slope of the contrast response function of the normalization (attention term was applied to the slope of the exponential function), but a multiplicative scaling of the normalization contrast function could explain the data virtually as well. Existing data cannot distinguish between variants such as these. Future experiments should provide data that reveal the precise relationship between attention and the normalization. Similarly, the current formulation does not allow attention to affect spontaneous activity, although this effect has been observed in many experiments [15], [26], [27]. Existing neurophysiological data do not require the model to include this component, because spontaneous activity is weak comparing to evoked response. It is difficult to obtain precise data on modulations of spontaneous activity owing to the low rates of firing involved, but it may be possible to refine the model with experimental data in the future.

### Concluding comments

While the simulations and neurophysiology presented here show that the attentional normalization model can help explain how attention operates in visual neurons, many questions remain to be addressed. It will be important to see if it can survive more extensive neurophysiological tests, in particular including data from neurons in the ventral pathway in visual cortex, such as those in area V4, as well as neurons in other sensory modalities and higher cortical areas. Also, it will be important to see if a correlation between response normalization and attention can be seen not only across neurons but also within individual neurons across different stimulus conditions. Finally, it will be important to see how readily the attentional normalization model can account for the effects of feature attention. While the attentional normalization model will undoubtedly need to be extended and refined, its success in explaining the range of phenomena described here suggest that it will prove useful in exploring and understanding the neuronal mechanisms of attention.

## Methods

All the procedures we used involving animals were approved by the Institutions Animal Care and Use Committees of Harvard Medical School or Baylor College of Medicine. Some of the neurophysiological data presented here have been described previously in the context of different observations (Lee and Maunsell, submitted).

### Animal preparation and behavioral task

We implanted a head post and scleral search coil on a rhesus monkey (*Macaca mulatta*, male, 8 kg) under general anesthesia. After recovery from the surgery, the animal was trained on a speed change detection task. During each trial the animal was required to hold its gaze within ±1° of a small spot at the center of a video display (44°×34°, 1024×768 pixels, 85 Hz, gamma-corrected), while series of achromatic Gabor stimuli were flashed synchronously in two or three locations on a gray background (42 cd/m^2^). Each set of Gabors was presented for 200 ms and successive sets were separated by intervals that varied randomly between 141 and 294 ms. The animal's task was to detect when a Gabor with a faster drift rate (the target) appeared in the cued location and to make a saccade to that location within 600 ms of its appearance. Correct responses were rewarded with a drop of water or juice. Speed changes also occurred in uncued locations (distractors), but responses to those changes terminated the trial without reward. The target location was cued at the start of each trial by a yellow annulus presented at that location for 300 ms. During recording from each neuron, the Gabors in all locations had the same standard deviation (σ), spatial frequency, and temporal frequency (except for targets and distractors).

The time in the trial when the target stimulus appeared followed an exponential distribution (a flat hazard function for speed change) in order to encourage the animal to keep its vigilance constant throughout each trial. However, if a trial reached 5 s without a target appearing (∼10% of trials) it was terminated and the animal was given a reward.

### Attentional modulation

To measure the effects of attention on neuronal responses, the task was performed using two series of Gabors. Pairs of Gabors were flashed in the receptive field of the neuron being recorded, and oriented so that Gabors in one position always drifted in the neuron's preferred direction while Gabors in the other position always drifted in the opposite, null direction. The two locations were equally eccentric from the fixation point. The positions and directions for the Gabors were determined using a separate quantitative receptive field mapping (see below). In this version of the task, pairs of Gabors always had the same contrast, but the contrast used for each presentation was randomly selected from 8 values (0, 1.56, 3.13, 6.25, 12.5, 25, 50, 100%). This allowed us to measure responses over a range of contrasts with attention directed either to a preferred- or a non-preferred stimulus in the receptive field.

### Stimulus interactions

To measure stimulus interactions, the same task was performed using three series of Gabors. Two were the Gabors used in the attentional modulation experiment, which were in the neuron's receptive field and moved in the preferred- and non-preferred directions. A third series was located at the same eccentricity in the opposite hemifield and moved in an orthogonal direction. In this task the animal's attention was always directed to the location outside the receptive field. The two Gabors in each pair in the receptive field always had contrasts that differed by a factor of two. In half of the presentations the Gabor drifting in the preferred direction had higher contrast, and in half of the presentations the null Gabor had higher contrast. The contrasts for each pair were randomly selected from 8 pair of values (0,0; 0.78,1.6; 1.6,3.1; 3.1,6.3; 6.3,12.5; 12.5,25; 25,50; 50,100%). The contrast of the Gabor outside the receptive field was randomly selected to match the contrast of one of the stimuli in the receptive field.

### Neurophysiological recording and analysis

After training was completed, we implanted a recording chamber over MT. We recorded the activity in single units using conventional extracellular techniques, which have been described in detail previously [15]. Once we isolated the action potentials of a neuron, we plotted the receptive field using hand-controlled visual stimuli. We then used computer-controlled presentations of Gabors to measure the neuron's tuning for direction of motion (12 directions), spatial frequency (10 frequencies), and temporal frequency (10 frequencies). We also mapped the receptive field quantitatively using a Gabor stimulus with the preferred direction of motion, spatial frequency, and temporal frequency (3 eccentricities by 8 polar angles). This mapping was used to select two isoeccentric receptive field locations that gave approximately equal responses.

Some parameters were estimated by fitting functions to the neuronal responses. For fitting data, we used a weighted-least square fit, where the variance of the measurement served as the weight. We used a von Mises distribution [50], [51] to estimate the direction tuning of each neuron:
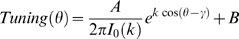
(6)


In this equation, 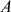 is a scaling factor, 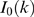 is the zeroth order Bessel function, 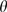 is the direction of motion, 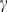 is the preferred direction of motion, and 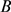 is spontaneous activity. We calculated half-width at half-height (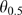) [50], [51] as:
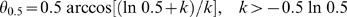
(7)


To measure normalization in stimulus interaction experiment and attention experiment, we estimated the CRFs of each neuron using a hyperbolic-ratio function [24]:
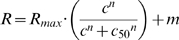
(8)


In this equation, 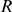 is the response of a cell, 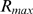 is the maximum attainable response of the cell (above spontaneous activity), 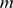 is the spontaneous activity, 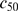 is the contrast where the response is half maximal, 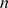 is the steepness of the function, and 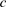 is the contrast of stimulus. When fitting data from the two attentional states in the attention experiments (attend preferred or attend null) or the two stimulus conditions in the stimulus interaction experiment (higher contrast on preferred or null), we used the same values of 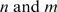 for both conditions and let 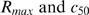 vary between conditions. The effect of task conditions on the responses at the upper saturation of the CRFs was calculated using a modulation index for 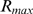:
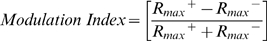
(9)where 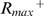 is 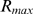 of one experimental condition (attend to preferred or higher contrast on preferred), and 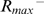 is 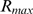 of the other (attend to null or higher contrast on null). This modulation index was used for all correlation analyses. We calculated 95% confidence intervals for these modulation indices using a bootstrap analysis (1000 resamplings). For the directionality calculation, we took a peak (the response to the preferred) and an offset (the response to the null) from the direction tuning function (Eq. 6) to compute the modulation index. Here, 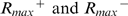 of Eq. 9 are replaced by the peak and offset. In some cases we used the response to the preferred stimulus from receptive field mapping testing instead of the peak of the direction tuning function.

### Attentional normalization model

We estimated the direct inputs to a cell using Eq. 3 and setting the contrast of the second stimulus to zero, which reduced the activity associated with that stimulus to spontaneous activity. This reduced Eq. 3 to following equation:
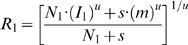
(10)in which 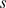 is the spontaneous activity of the normalization (Eq. 4) and 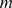 is the spontaneous activity of the neuron. The two direct inputs where therefore:
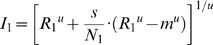
(11)

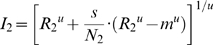
(12)


These direct inputs were established in the no-attention condition, so the normalization term 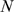 has 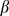 fixed to 1.

In the attentional normalization model, responses to individual stimuli could be determined from empirical observation. However, we did not directly measure 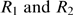. Instead, we approximated them by estimating the CRF for each neuron. For this, we used a hyperbolic ratio function (Eq. 8). The spontaneous activity of each neuron, 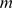, was taken from the response to zero contrast stimuli in stimulus interaction measurements. 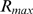 for the preferred and null stimulus was based on response from direction tuning functions (Eq. 6). The slope, 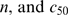 of the CRF were taken from the CRFs from the attention experiment. We used 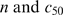 from the attention experiment because 1) contrast of stimuli at a given contrast were the same here and 2) the previous study reported that attention does not effectively shift the CRF of MT neuron (Lee and Maunsell, submitted). Errors introduced by the indirect determination of 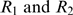 should not bias the outcome of the model fits.

### Simulation for response summation

We simulated contrast dependent response summation using the attentional normalization model. We used a hyperbolic ratio function (Eq. 8) to make CRFs for 100 neurons with a single stimulus in the receptive field. For each simulated neuron, we drew two values of 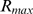, one for a central receptive field location and one for a flanking location in the receptive field. Each 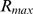 was drawn from a Gaussian distribution, using a mean and SD of 100 and 20 spikes/s for the central location and 30 and 6 spikes/s for the flanking location. Similarly, we drew 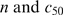 from second order Gamma distributions with means of 3.6 and 0.21, which are median values of measured values for MT neurons [23].

To make pair responses, we used the attentional normalization model (Eq. 3) and drew the three parameters of the model (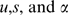) from second order Gamma distributions with means of 3.4, 0.05, and 0.2, which were the mean values estimated by fitting the physiological data from the attention and stimulus interaction experiments. For each neuron we produced four sets of responses to pair and individual stimuli, in which the sets were obtained by varying location of stimuli in the receptive field (two central locations, two flank locations, a central location for stimulus 1 and a flank location for stimulus 2, and a central location for stimulus 2 and a flank location for stimulus 1).

### Simulations for attention

We simulated single or paired stimuli by setting the contrast of the second stimulus to zero or one (Eq. 3). To make direction tuning functions, we used 90 spikes/s for the response to the preferred stimulus, 15 spikes/s for the response to the null stimulus, and 60 degrees for the half-width at half-height of the tuning function. The tuning function was made from a Gaussian function, where the scaling factor and the offset of the function were the preferred and the null responses respectively:
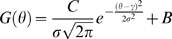
(13)where 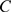 is a scaling factor, 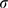 is the standard deviation of the function, 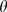 is the direction of motion, 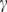 is the preferred direction of motion, and 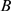 is the offset of the function. Here we made 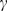 zero and calculated 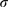 from the half-width of the tuning:
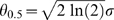
(14)where 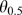 is half-width at half-height.

We simulated the effect of attention on the direction tuning function using the attentional normalization model. We used each response at each direction of motion for deriving direct inputs (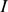), and simulated the tuning function when attention was directed to the stimulus in the receptive field. The simulation for the responses to a pair of stimuli was done using the same model parameters. Here, we only used the scaling factor (the preferred response) and the offset (the null response) of the direction tuning function (Eq. 13) for deriving direct inputs, and simulated a pair response to the two stimuli and the effect of attention.
